# Cadmium Is Accumulated as Electron-Dense Nanoparticles, Not Bound to Glutathione (GSH), Phytochelatins or Metallothioneins, and Extruded to the Culture Medium with GSH in the Marine Alga *Ulva compressa*

**DOI:** 10.3390/ijms27125608

**Published:** 2026-06-22

**Authors:** Paulina Cabezas, Stephanie Romero, Patricia Méndez, Bryan Pichún, Rodrigo Segura, Héctor Osorio, Alberto González, Alejandra Moenne

**Affiliations:** 1 Laboratory of Marine Biotechnology, Faculty of Chemistry and Biology, University of Santiago of Chile, Alameda 3363, Santiago 917022, Chile; paulina.cabezas@usach.cl (P.C.); stephanie.romero@usach.cl (S.R.); patricia.mendez@usach.cl (P.M.); hector.osorio@usach.cl (H.O.); alberto.gonzalezfi@usach.cl (A.G.); 2 Laboratory of Inorganic Chemistry, Faculty of Chemistry and Biology, University of Santiago of Chile, Alameda 3363, Santiago 917022, Chile; bryan.pichun@usach.cl (B.P.); rodrigo.segura@usach.cl (R.S.); 3 Institute on Green Ammonia as Energy Vector-MIGA, Santiago 7820436, Chile

**Keywords:** cadmium, extrusion, glutathione, phytochelatins, metallothioneins, sulfide donor, marine alga, *Ulva compressa*

## Abstract

The mechanism of cadmium (Cd) accumulation was analyzed in the marine alga *Ulva compressa*. The alga was cultivated with 10 µM Cd, with 10 µM of Cd and increasing concentrations of a sulfide donor (NaHS), or with a sulfide acceptor (hypotaurine), and intracellular Cd levels were monitored for 7 d. Glutathione (GSH) and phytochelatins (PCs) levels, and metallothioneins (MTs) transcript levels were also quantified, along with the extrusion of Cd, GSH, and PCs to the culture medium. The results showed that the sulfide donor increased intracellular Cd levels, whereas the sulfide acceptor decreased them. GSH, PCs, and MTs levels did not correlate with intracellular Cd contents. Both Cd and GSH were extruded to the culture medium, along with lower amounts of PCs. TEM-EDXS analysis revealed electron-dense nanoparticles containing Cd and O, likely CdO or Cd bound to fatty acids; in the presence of NaHS, nanoparticles containing Cd and S (likely CdS) or Cd, S, and N (likely Cd bound to GSH) were also observed. In conclusion, Cd accumulates as insoluble nanoparticles—probably not bound to GSH, PCs, or MTs—and is extruded to the culture medium together with GSH in the marine alga *U. compressa*.

## 1. Introduction

Cadmium (Cd) is a non-essential heavy metal that is not required for the activity of any enzyme, except a carbonic anhydrase of marine diatoms, in which the metal can act as a cofactor replacing Zn [1]. Cd has a hydrated radius similar to zinc and, thus, can also replace zinc in plant enzymes, inactivating them [2]. In plants, Cd induces the synthesis of glutathione (GSH) and phytochelatins (PCs), which are formed by condensed units of GSH and synthesized by the enzyme phytochelatin synthase (PCS). Cd also induces the expression of metallothioneins (MTs), which are small proteins (<10 kDa) rich in cysteine residues (around 30% of total amino acids). In plants, GSH, PCs, and MTs chelate Cd ions through the sulfhydryl groups of cysteine residues and Cd bound to GSH and/or PCs can be stored in plant vacuoles [3].

Regarding Cd accumulation in vascular plants, it has been shown that overexpression of PCS from the aquatic plant *Ceratophyllum demersum* in tobacco plants enabled the accumulation of Cd and arsenic [4]. It has also been shown that *A. thaliana* cultivated with 50 µM of Cd showed a sustained increase in H_2_S due to the increased expression of the enzymes L-cysteine desulfhydrase (CD) and O-acetyl thiol lyase, which also has CD activity (DES1), both involved in cysteine degradation and the production of ammonia and H_2_S in roots [5]. In addition, the increase in H_2_S leads to increased expression of enzymes that synthesize cysteine; the increase in cysteine content enhances the expression of enzymes involved in GSH synthesis; and the increase in GSH level enhances the expression of the PCS enzyme, which synthesizes PCs [5]. Furthermore, the legume *Trifolium repens* cultivated hydroponically with 300 µM Cd for 7 d showed an increased expression of genes encoding enzymes involved in cysteine and GSH synthesis and PCS, which synthesizes PCs in roots and leaves [6]. On the other hand, rice MT1e overexpression increased Cd accumulation, mainly in the shoots, and MT1e mutation reduced Cd accumulation in the shoots [7]. Furthermore, *A. thaliana* cultivated with 50 µM of Cd showed an increased expression of MT1a, which may be involved in Cd chelation and/or ROS scavenging [8]. Thus, Cd is accumulated in plants through binding with GSH, PCs, and MTs.

Regarding Cd accumulation in non-vascular plants, Cd is accumulated in the vacuole of the liverwort *Marchantia polymorpha*, and it is also extruded to the culture medium complexed with GSH and PCs [9]. Furthermore, the mutation of PCS in *M. polymorpha* decreased Cd tolerance but did not decrease tolerance to other heavy metals, such as zinc, iron, nickel, and arsenic. Moreover, the overexpression of PCS increased Cd accumulation [10]. Interestingly, *M. polymorpha* is an ancient non-vascular plant, and PCs synthesis responds only to Cd, not to other heavy metals, suggesting that the synthesis of GSH and PCs represents an ancient mechanism to avoid Cd toxicity in non-vascular plants [10]. Thus, in non-vascular plants, Cd is accumulated through binding to GSH and PCs.

Regarding Cd accumulation in green microalgae, in the microalga *Chlamydomonas* sp. cultivated with Cd and analyzed by transmission electron microscopy (TEM) coupled to energy-dispersive X-ray spectroscopy (EDXS), Cd was accumulated as electron-dense particles containing Cd, probably in fragmented vacuoles [11]. In the green microalga *C. acidophila*, Cd was accumulated as electron-dense particles containing Cd, probably also in fragmented vacuoles [12]. In the green microalga *Scenedesmus* sp., Cd was accumulated as electron-dense particles containing Cd in mitochondria and chloroplasts, probably bound to fatty acids [13]. In addition, the green microalga *Micrasterias denticulata* (Streptophyta) accumulates Cd as CdO [14] and synthesizes PCs in response to Cd, mainly PC2 and PC3 [15]. Thus, in green microalgae, Cd is probably accumulated as electron-dense particles bound to fatty acids or as CdO.

In plants, Cd is transported into the roots through iron/zinc transporters IRT1, IRT2, and IRT3 [16,17,18] and through zinc, iron, and manganese transporters NRAMP1 and ZIP2 [19,20,21]. Then, Cd is transported into the xylem and to the shoots by the ATP-dependent transporters HMA2 and HMA4 [22,23]. In addition, HMA1 and HMA3 transport Cd bound to GSH to the vacuole, and ABCC1 and ABCC2 transport Cd bound to PCs to the vacuole [24,25,26]. In this regard, *Brassica chinensis* cultivated with 20 and 100 µM of Cd and sprayed on leaves with GSH showed an increased expression of IRT1, IRT2, HMA2, and HMA3 transporters [27]. A transcriptomic analysis of *T. repens* cultivated hydroponically with 300 µM of Cd for 7 d showed that 40 Cd transporters were overexpressed in roots and leaves, such as ZIP transporters, Yellow Stripe 1-like (YSL) transporters, multidrug resistance (MDR) proteins, multidrug extrusion (MATE) proteins, and ATP-binding cassette (ABC) transporters, HMA, and CTR [6].

The marine alga *Ulva compressa* is a cosmopolitan green alga that, when cultivated with 10 µM of copper or Cd, showed intracellular accumulation of both heavy metals [28,29,30,31]. It was initially proposed that copper can be accumulated in the alga bound to GSH, PCs, and/or MTs, as in plants [28]. However, it has recently been shown that copper accumulates in *U. compressa* as copper sulfide particles in the chloroplast, rather than being bound to GSH, PCs, or MTs [31]. To this end, the sulfide donor NaHS and the sulfide acceptor hypotaurine were used, and intracellular copper levels were determined [32]. It was shown that copper accumulation increases in response to the sulfide donor and decreases with the sulfide acceptor, suggesting that copper is accumulated as copper sulfide. In addition, analyses using TEM-EDXS showed that copper in response to a sulfur donor was accumulated as electron-dense particles containing copper and sulfur, but not N, in the cytosol and chloroplasts [32]. Thus, copper is accumulated as copper sulfide in the marine alga *U. compressa*.

In this work, we analyzed the accumulation of Cd in *U. compressa* in response to 10 µM of Cd and 0, 50, 100, and 200 µM NaHS for 0 to 7 d, or with 10 µM Cd of Cd and 500 µM of hypotaurine for 5 d, and determined the levels of GSH and PCs and the expression of MTs. We also analyzed Cd extrusion with GSH and/or PCs and visualized electron-dense Cd-containing nanoparticles using TEM-EDXS. The aim of this study is to determine the mechanism of cadmium accumulation, to compare with cadmium accumulation in response to a sulfur donor or sulfur acceptor, and to compare with mechanisms involved in copper accumulation previously described [32].

## 2. Results

### 2.1. Intracellular Accumulation of Cd in Response to NaHS or Hypotaurine

**Figure 1:**
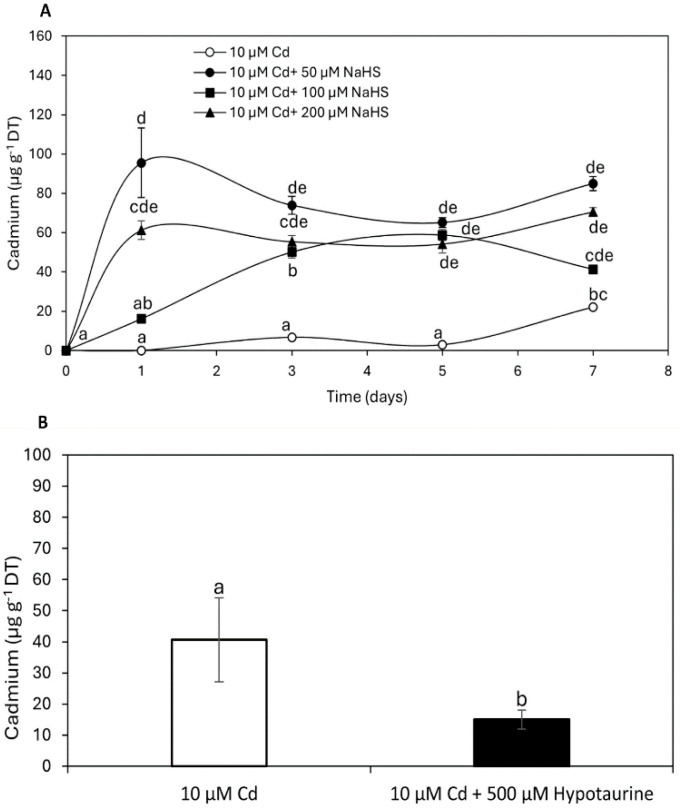
Level of intracellular cadmium in the alga *U. compressa* cultivated with 10 µM of cadmium (open circles), with 10 µM of cadmium and 50 µM of NaHS (black circles), with 10 µM of cadmium and 100 µM of NaHS (black square), or with 10 µM of cadmium and 200 µM of NaHS (black triangle) for 0 to 7 days (**A**), and in the alga cultivated with 10 µM of cadmium with 500 µM of hypotaurine for 5 days (**B**). The level of intracellular cadmium is expressed in micrograms per gram of dry tissue (DT). Symbols (**A**) and bars (**B**) represent mean values of three independent experiments ± SD. Letters indicate significant differences (*p* < 0.05).

The alga cultivated in artificial seawater without Cd did not show accumulation of Cd in its tissue for 0 to 7 d. The alga cultivated with 10 µM of Cd showed a slight increase in Cd only at day 7 (Figure 1A). The alga cultivated with 10 µM of Cd and 50 µM of NaHS showed a significant increase at day 1 that remained unchanged until day 7. The alga cultivated with 10 µM of Cd and 100 µM of NaHS showed a sustained increase until day 3 that remained unchanged until day 7. The alga cultivated with 10 µM of Cd and 200 µM of NaHS showed a significant increase at day 1 that remained unchanged until day 7 (Figure 1A, Supplementary Table S1). Thus, the sulfide donor NaHS induced an increase in Cd intracellular accumulation in *U. compressa*.

In contrast, adult algae cultivated with 10 µM of Cd and 500 µM of hypotaurine for 5 d showed a significant decrease in Cd level (Figure 1B). Thus, the sulfide acceptor hypotaurine decreased the level of intracellular Cd, suggesting that Cd can be accumulated, at least in part, as CdS.

### 2.2. Level of GSH and PCs in Response to Cd and NaHS

**Figure 2:**
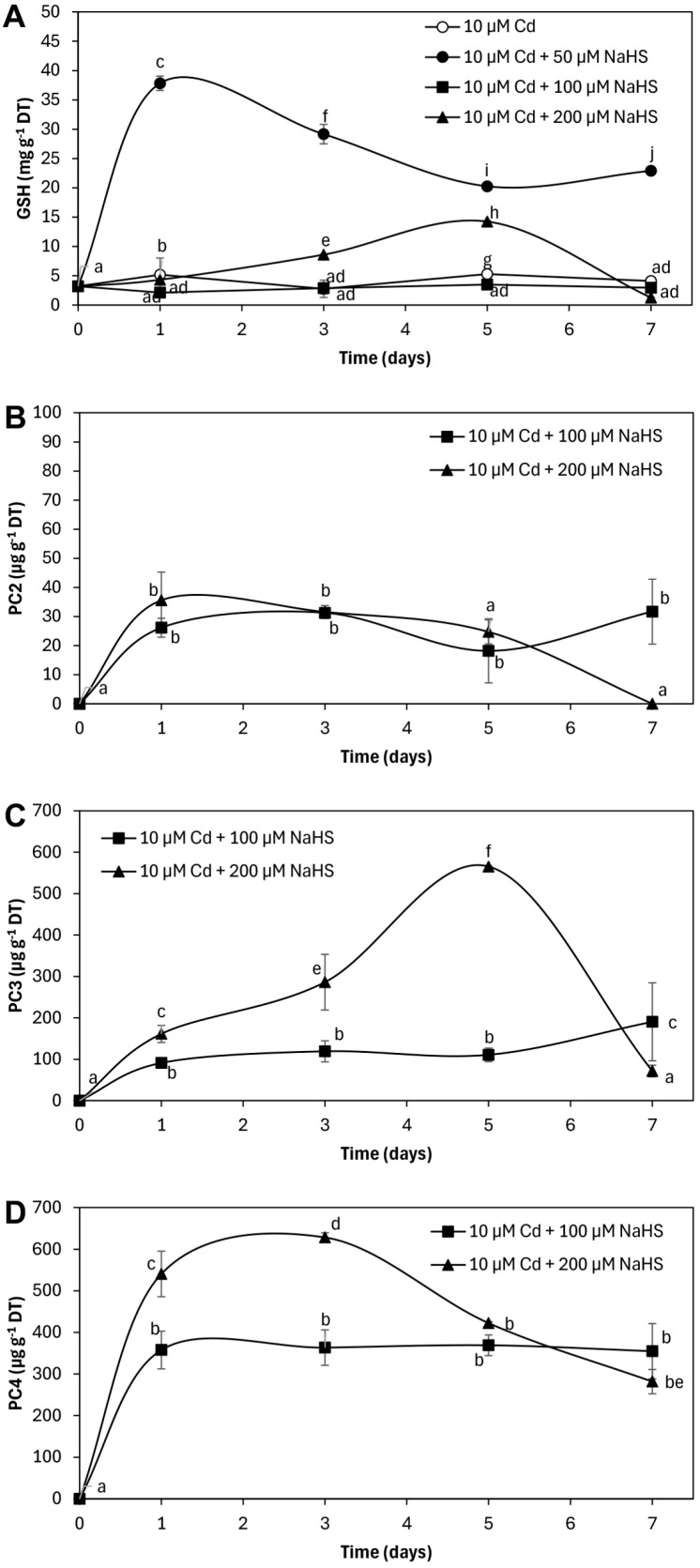
Level of glutathione (GSH) and phytochelatins (PCs) in the marine alga *U. compressa* cultivated with 10 µM of cadmium (open circles), with 10 µM of cadmium and 50 µM of NaHS (black circles), with 10 µM of cadmium and 100 µM of NaHS (black square), or with 10 µM cadmium and 200 µM of NaHS (black triangle) for 0 to 7 days (**A**–**D**). GSH and PCs are expressed in micrograms per gram of dry tissue (DT). Symbols represent mean values of three independent experiments ± SD. Letters indicate significant differences (*p* < 0.05).

The level of GSH in the alga cultivated with 10 µM of Cd slightly increased at day 1, decreased at day 3, slightly increased at day 5 and decreased to control level at day 7 (Figure 2A). The level of GSH in the alga cultivated with 10 µM of Cd and 50 µM of NaHS significantly increased at day 1 and decreased until 7 (Figure 2A, Supplementary Table S2). The level of GSH in the alga cultivated with 10 µM of Cd and 100 µM of NaHS did not show an increase from day 1 to day 7 (Figure 2A). The level of GSH in the alga cultivated with 10 µM of Cd and 200 µM of NaHS showed a sustained increase until day 5 that decreased to control level at day 7 (Figure 2A). Thus, the level of GSH did not change in the algae cultivated only with Cd or with Cd and 100 µM of NaHS, but the level of Cd increased in the alga cultivated with Cd and 50 µM and 200 µM of NaHS. Thus, the level of GSH did not correlate with the level of intracellular Cd, since intracellular cadmium increased with 5, 100 and 200 µM of NaHS, but GSH mostly increased with 50 µM of NaHS and much less with 100 and 200 µM of NaHS.

The levels of PC2, PC3, and PC4 were below the detection limit in the algae cultivated without Cd, with 10 µM of Cd, or with 10 µM of Cd and 50 µM of NaHS (Figure 2B–D). In contrast, the level of PC2 in the alga cultivated with 10 µM of Cd and 100 µM of NaHS increased at day 1 and remained increased until day 7, and in the alga cultivated with 10 µM of Cd and 200 µM of NaHS the level of PC2 increased at day 1, remained increased until day 5 and decreased at day 7 (Figure 2B, Supplementary Table S3). The level of PC3 in the alga cultivated with 10 µM of Cd and 100 µM of NaHS increased at day 1 and remained unchanged until day 7, and in the alga with 10 µM of Cd and 200 µM of NaHS it increased gradually until day 5 and decreased at day 7 (Figure 2C, Supplementary Table S4). The level of PC4 in the alga cultivated with 10 µM of Cd and 100 µM of NaHS increased at day 1 and remained unchanged until day 7, and in the alga with 10 µM of Cd and 200 µM of NaHS the level of PC4 increased at day 1, increased again at day 3 and decreased at day 5 and 7 (Figure 2D, Supplementary Table S5). Thus, the levels of PC3 and PC4 were the most increased compared to PC2 level, and the increases in PC3 and PC4 were higher in response to 200 µM of NaHS compared to 100 µM of NaHS.

### 2.3. Expression of MT1.1 Family in Response to Cd and NaHS

**Figure 3:**
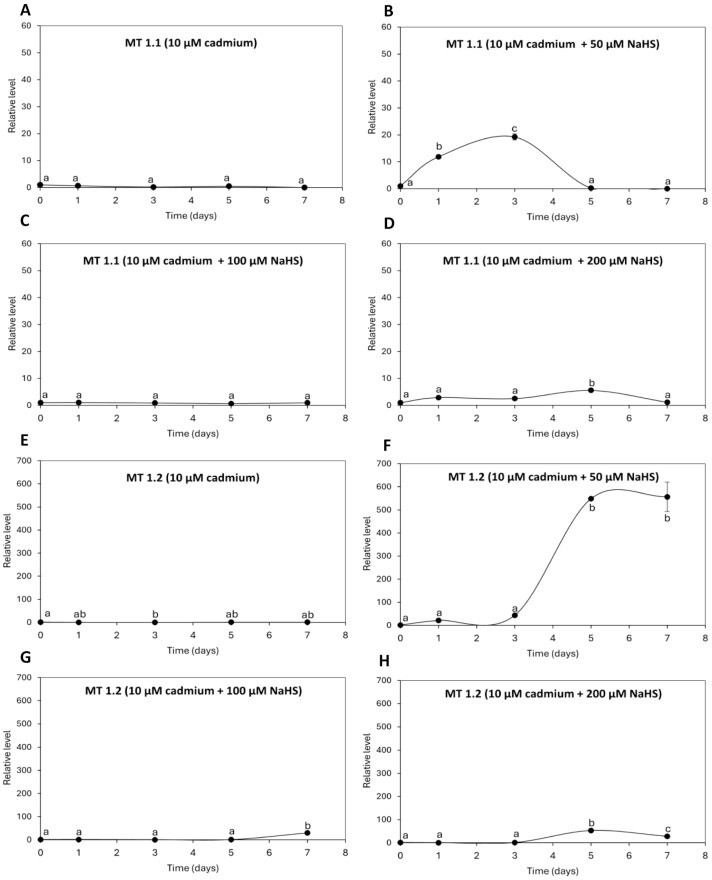
Relative levels of transcripts encoding *U. compressa* metallothioneins (MTs) belonging to the MT1.1 family, corresponding to UcMT1.1 and UcMT1.2, in the alga treated with 10 µM of cadmium (**A**,**E**), with 10 µM of cadmium and 50 µM of NaHS (**B**,**F**), 10 µM of cadmium and 100 µM of NaHS (**C**,**G**), and 10 µM of cadmium and 200 µM of NaHS (**D**,**H**). Symbols represent mean values of three independent experiments ± SD. Letters indicate significant differences (*p* < 0.05).

The relative level of transcripts encoding MT1.1 did not change in the algae cultivated with 10 µM of Cd or with 10 µM of Cd and 100 µM of NaHS (Figure 3A–C; Supplementary Table S6). The level of transcripts encoding MT1.1 in the alga cultivated with 10 µM of Cd and 50 µM of NaHS increased 19 times at day 3 and decreased to control level at day 5 and 7 (Figure 3B). The level of transcripts encoding MT1.1 in the alga cultivated with 10 µM of Cd and 200 µM of NaHS increased 6 times at day 5 and decreased to control level at day 7 (Figure 3D). Thus, the level of MT1.1 only increased with 50 and 200 µM of NaHS, but not with 100 of NaHS, and the latter does not correlate with the level of intracellular cadmium.

On the other hand, the level of transcripts encoding MT1.2 did not increase with 10 µM of Cd or with 10 µM of Cd and 100 µM of NaHS, as observed for MT1.1 (Figure 3E–G; Supplementary Table S7). The level of transcripts encoding MT1.2 in the alga cultivated with 10 µM of Cd and 50 µM of NaHS increased 43 times at day 3, increased 548 times at day 5, and remained unchanged at day 7 (Figure 3F). The level of transcripts encoding MT1.2 in the alga cultivated with 10 µM of Cd and 200 µM of NaHS increased 53 times at day 5 and decreased at day 7 (Figure 3H). Thus, the level of MT1.2 only increased with 50 µM of NaHS and slightly increased with 200 µM of NaHS, and this does not correlate with the levels of intracellular cadmium.

**Figure 4:**
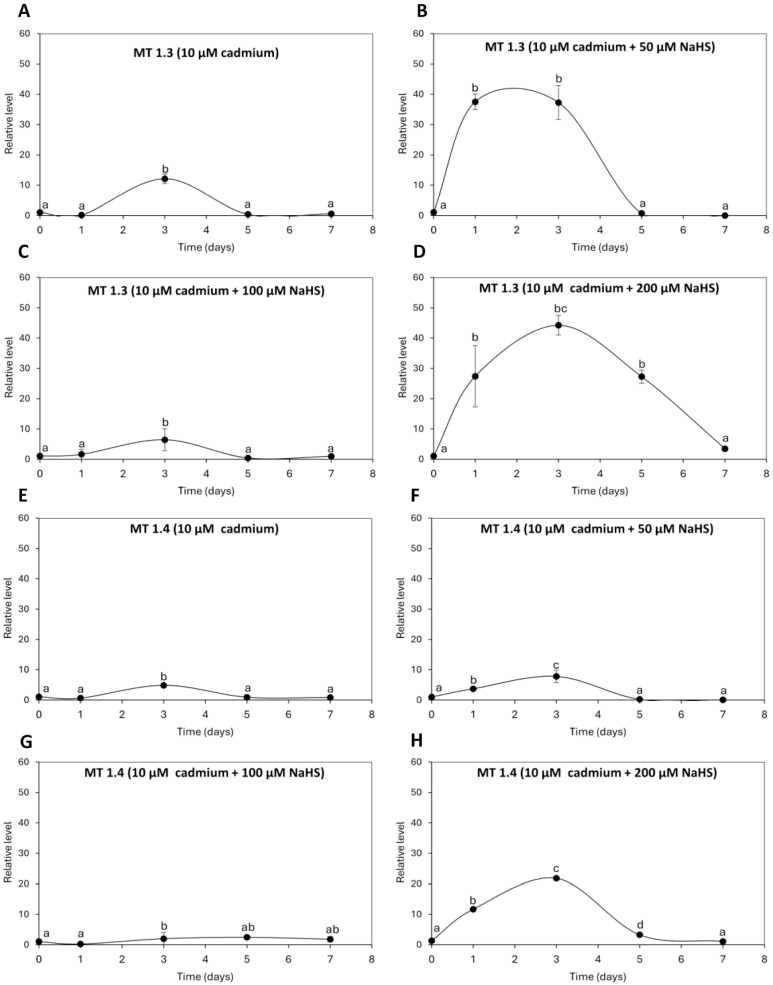
Relative levels of transcripts encoding *U. compressa* metallothioneins (MTs) belonging to the UcMT1.1 family, corresponding to MT1.3 and MT1.4, in the alga treated with 10 µM of cadmium (**A**,**E**), with 10 µM of cadmium and 50 µM of NaHS (**B**,**F**), 10 µM of cadmium and 100 µM of NaHS (**C**,**G**), and 10 µM of cadmium and 200 µM of NaHS (**D**,**H**). Symbols represent mean values of three independent experiments ± SD. Letters indicate significant differences (*p* < 0.05).

The level of transcripts encoding MT1.3 in the alga cultivated with 10 µM of Cd increased 12 times at day 3 and decrease to control level at days 5 and 7 (Figure 4A, Supplementary Table S8). The level of transcripts in the alga cultivated with 10 µM of Cd and 50 µM of NaHS increased 37 times at day 1 and 3 and decreased to control level at days 5 and 7 (Figure 4B). The level of transcripts encoding MT1.3 in the alga cultivated with 10 µM of Cd and 100 µM increased 6 times at day 3 and then decreased to control level at days 5 and 7 (Figure 4C). The level of transcripts encoding MT1.3 in the alga cultivated with 10 µM of Cd and 200 µM of NaHS increased 44 times at day 3 and decreased to control level at day 7 (Figure 4D). Thus, the level of MT1.3 increased with 50 and 200 µM of NaHS and only slightly increased with 100 µM of NaHS, and the latter does not correlate with the intracellular levels of cadmium.

On the other hand, the level of transcripts encoding MT1.4 in the alga cultivated with 10 µM of Cd increased 5 times at day 3 and decreased to control level at days 5 and 7 (Figure 4E, Supplementary Table S9). The level of transcripts encoding MT1.4 in the alga cultivated with 10 µM of Cd and 50 µM of NaHS increased 8 times at day 3 and decreased to control level at days 5 and 7 (Figure 4F). The level of transcripts encoding MT1.4 in the alga cultivated with 10 µM of Cd and 100 µM of NaHS increased 2.4 times at day 5 and decreased to 1.8 times at day 7 (Figure 4G). The level of transcripts encoding MT1.4 in the alga cultivated with 10 µM of Cd and 200 µM of NaHS increased 22 times at day 3 and decreased to the control level at day 7 (Figure 4H). Thus, the level of transcripts of MT1.4 only slightly increased with 50, 100 or 200 µM of NaHS, and the latter did not correlate with the intracellular levels of cadmium.

Therefore, higher increases in MT1 family transcript levels were observed for MT1.2 and MT1.3 in response to Cd and 50 and 200 µM NaHS, mainly for MT1.2 that only increased with 50 µM of NaHS.

### 2.4. Expression of MT2, but Not MT3, in Response to Cd and NaHS

**Figure 5:**
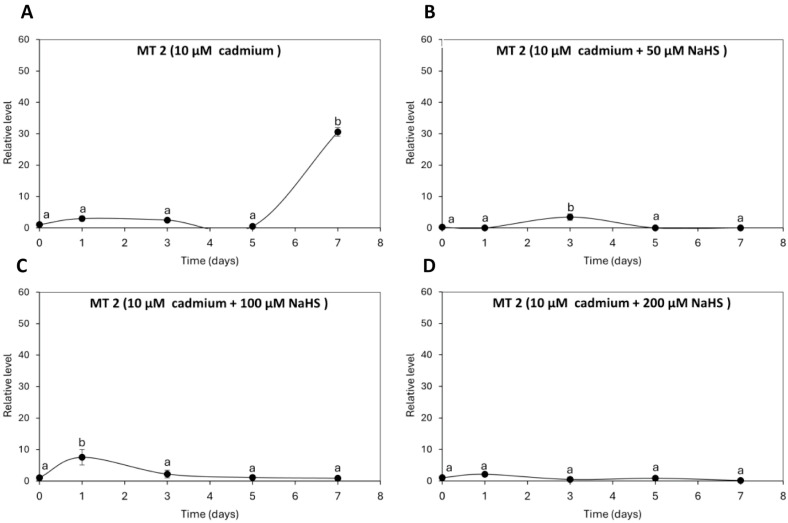
Relative level of transcripts encoding *U. compressa* metallothionein MT2 in the alga treated with 10 µM of cadmium (**A**), with 10 µM of cadmium and 50 µM of NaHS (**B**), 10 µM of cadmium and 100 µM of NaHS (**C**), and 10 µM of cadmium and 200 µM of NaHS (**D**). Symbols represent mean values of three independent experiments ± SD. Letters indicate significant differences (*p* < 0.05).

The level of transcripts encoding MT2 increased 31 times at day 7 in response to 10 µM of Cd (Figure 5A, Supplementary Table S10). The level of transcripts encoding MT2 in the alga cultivated with 10 µM of Cd and 50 µM of NaHS increased 3.4 times at day 3 and decreased to control level at days 5 and 7 (Figure 5B). The level of transcripts encoding MT2 in the alga cultivated with 10 µM of Cd and 100 µM of NaHS increased 8 times at day 1 and decreased to control level at days 5 and 7 (Figure 5C). The level of transcripts encoding MT2 in the alga cultivated with 10 µM of Cd and 200 µM of NaHS increased 2 times at day 1 and decreased to control level at days 3, 5 and 7 (Figure 5D). In contrast, the level of transcripts encoding MT3 did not increase in response to Cd, or Cd with increasing concentrations of NaHS. Thus, the level of transcripts encoding MT2 was lower than the level of transcripts encoding MT1.1 and MT1.2, whereas MT3 did not respond to Cd or Cd with NaHS. The increase in transcripts encoding MT2 did not correlate with Cd accumulation, since the increase in MT2 expression was low in response to Cd and 50 µM of NaHS, which is not in accord with the previously observed increased accumulation of Cd in response to Cd and 50 µM of NaHS.

### 2.5. Extrusion of Cd with Glutathione to the Culture Medium

**Figure 6:**
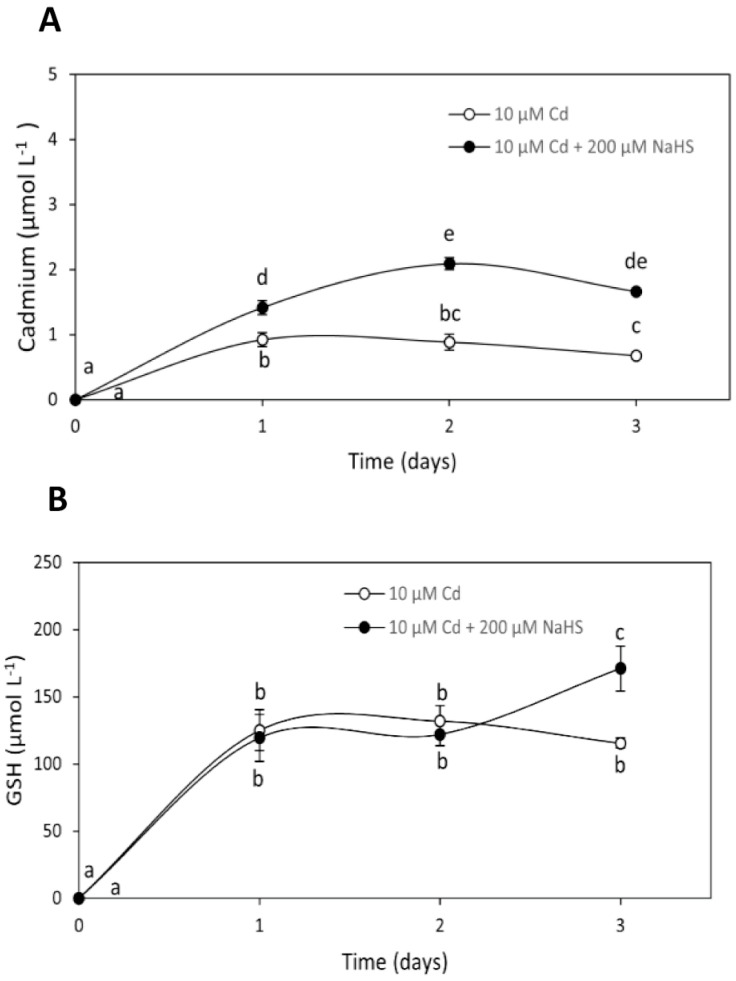
Level of cadmium (**A**) and glutathione (GSH) in the culture medium (**B**) in the alga *U. compressa* cultivated with 10 µM of cadmium (open circles) and with 10 µM of cadmium and 200 µM of NaHS (black circles). The concentration of cadmium and GSH is expressed in micromoles per liter. Symbols represent mean values of three independent experiments ± SD. Letters indicate significant differences (*p* < 0.05).

The alga was cultivated with 10 µM Cd or with 10 µM Cd and 200 µM NaHS for 5 days, then transferred to a Cd-free culture medium. Samples were collected at 0, 1, 2, and 3 d of culture, and Cd, as well as the levels of GSH and PCs, were analyzed in the culture medium. The culture medium at day 0 did not contain Cd or GSH. The culture medium of the alga cultivated only with Cd showed an increase in Cd level at day 1 and 2 and remained unchanged at day 3 (Figure 6A). The culture medium of the alga cultivated with Cd and 200 µM NaHS showed a higher increase in Cd at days 1 and 2, which remained unchanged until day 3. Thus, the alga cultivated only with Cd extruded Cd to the culture medium, and this extrusion increased in response to NaHS.

The alga cultivated only with Cd showed that GSH levels in the culture medium increased at day 1 and remained unchanged until day 3. The alga cultivated with Cd and 200 µM of NaHS showed an increase in GSH level at day 1 and a second increase at day 7 (Figure 6B). Thus, GSH was extruded to the culture medium in response to Cd, and to Cd and NaHS, but the extrusion of GSH was higher with 200 µM of NaHS than without NaHS at day 7. In addition, the GSH level is much higher than the Cd level in the culture medium. It is important to mention that PC2, PC3, and PC4 were not present in the culture medium at day 0, and they were extruded to the culture medium in response to Cd, or Cd and 200 µM of NaHS, but their levels were very low (nmoles L^−1^) compared to Cd or GSH levels (µmoles L^−1^), which may indicate that extrusion of PCs is not related to Cd extrusion to the culture medium.

### 2.6. Accumulation of Cd as Electron-Dense Nanoparticles in U. compressa

**Figure 7:**
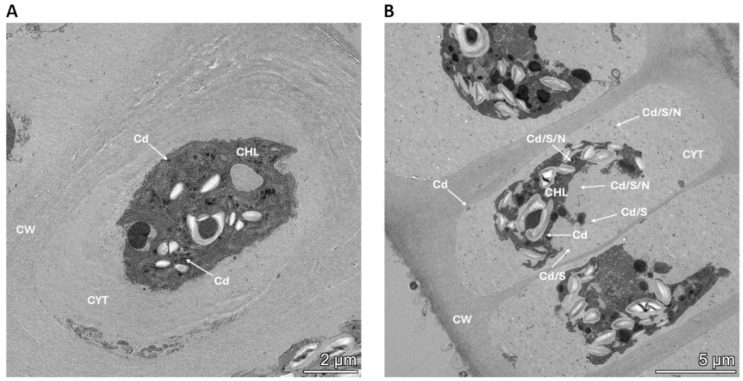
Visualization and analysis of electron-dense nanoparticles by TEM-EDX in the alga *U. compressa* cultivated with 10 µM of cadmium (**A**) and 10 µM of cadmium and 200 µM of NaHS (**B**) for 7 days. Arrows indicate cadmium (Cd) in the chloroplast (**A**) and Cd, Cd and sulfur (Cd/S), and cadmium, sulfur and nitrogen (Cd/S/N) in the cytosol (CYT) and in the chloroplast (CHL), but not in the cell wall (CW).

Regarding Cd accumulation in *U. compressa*, the alga treated only with Cd showed electron-dense nanoparticles that do not contain S or N. Still, it contained O and C, located in the chloroplast (Figure 7A). In contrast, the alga treated with Cd and NaHS showed electron-dense nanoparticles that contain S and/or N, and also O and C, and other nanoparticles that do not contain S or N, but contain O and C, in the cytosol and in the chloroplast (Figure 7B). Thus, Cd is accumulated in the chloroplast, probably bound to fatty acids or as CdO, and Cd with NaHS is accumulated in the cytosol and chloroplast, bound to S, as CdS, or bound to S and N, probably bound to GSH.

## 3. Discussion

In this work, we showed that Cd is accumulated intracellularly in the alga *U. compressa* and that the addition of a sulfide donor increased Cd accumulation, whereas a sulfide acceptor decreased Cd accumulation, suggesting that Cd could be accumulated as CdS in the alga treated with NaHS. In this regard, it has been previously shown that copper only accumulates as copper sulfide in *U. compressa*. Interestingly, the level of copper accumulated in the alga cultivated with 10 µM of copper reached 60–120 µg g^−1^ FW after 5 d of culture [31], whereas the alga cultivated with 10 µM of Cd accumulated only 22–41 µg g^−1^ FW after 5 d of culture (this work), suggesting that Cd might be more efficiently extruded from the alga compared to copper.

Regarding GSH and PCs in response to Cd in *U. compressa*, GSH levels increased in response to Cd and 50 µM NaHS but decreased at 100 and 200 µM NaHS. In addition, PC2, PC3, and PC4 increased in response to 100 and 200 µM NaHS, mainly PC3 and PC4. Thus, the higher level of GSH in the alga cultivated with 50 µM NaHS is consistent with the lack of PC synthesis in response to 50 µM NaHS and probably with a lower extrusion of Cd. The level of GSH was lower in response to 100 and 200 µM NaHS, which is in accord with the synthesis of PCs in response to 100 and 200 µM NaHS. The latter is consistent with the greater increase in intracellular Cd accumulation observed with 50 µM NaHS, suggesting that GSH may be required for Cd extrusion. In contrast, Cd accumulation decreased in response to 100 and 200 µM NaHS, and GSH levels decreased compared to 50 µM NaHS, suggesting that GSH and/or PCs may be required to extrude Cd. In this sense, it has been shown that in the liverwort *M. polymorpha*, Cd is extruded into the culture medium complexed with GSH and PCs [9]. *M. polymorpha* is an ancient non-vascular plant in which PCs are synthesized only in response to Cd, and not to other heavy metals, including copper. Thus, it was proposed that PC synthesis is an ancient mechanism to avoid Cd toxicity in plants [10], and this ancient mechanism was already present in marine green algae before the emergence of terrestrial plants.

Regarding MTs and Cd accumulation in *U. compressa*, MTs showed differential expression in response to Cd or Cd with NaHS, but this did not correlate with intracellular Cd accumulation. The most highly expressed MTs were MT1.1 and MT1.2, which were mainly upregulated in response to Cd and 50 µM NaHS; MT1.3 and MT1.4 mainly responded to 50 and 200 µM NaHS. The latter contrasts with results obtained with *U. compressa* exposed to copper or to copper and NaHS, which showed increases in the expression of MT1.1, MT1.2, MT1.3, and MT1.4 in response to copper and 50 and 100 µM NaHS, but not in response to copper and 200 µM NaHS [32]. In addition, the most highly expressed MT in response to copper was MT1.2 with 50 and 100 µM NaHS. Thus, similar micromolar concentrations of copper or Cd (10 µM) are differentially perceived, triggering differential responses in the synthesis of GSH and PCs and in the expression of MTs in the marine alga *U. compressa*.

Regarding Cd extrusion to the culture medium in *U. compressa*, the alga cultivated with 10 µM Cd extruded Cd to the culture medium complexed with GSH and, to a lesser extent, with PCs, and this phenomenon was increased by a sulfide donor. This result is consistent with the fact that Cd is accumulated in the liverwort *M. polymorpha* but is also extruded into the culture medium, complexed mainly with GSH [9]. In addition, the mutation of PCS in *M. polymorpha* decreased Cd tolerance but did not decrease tolerance to other heavy metals, such as zinc, iron, nickel, and arsenic. PC synthesis occurs only in response to Cd, not to other heavy metals, and it was proposed that PC synthesis is an ancient mechanism to protect plants against Cd toxicity [10]. Green marine algae are more ancient than terrestrial plants and, as shown in this work, green algae can also extrude Cd complexed with GSH and, to a lesser extent, with PCs to counteract Cd toxicity, indicating that Cd extrusion with GSH is a more ancient mechanism, as it is already present in marine green algae.

Regarding transporters that may be involved in Cd extrusion in *U. compressa*, it is important to mention that a transcriptomic analysis performed in *U. compressa* cultivated with 10 µM copper for 24 h detected the overexpression of transporters such as NRAMP, ZIP, IRT, HMA, MATE, YSL, ABCC, and OPT types, as has been described in terrestrial plants and green microalgae. Considering that Cd is extruded complexed with GSH into the culture medium, the Cd transporter responsible for extrusion could be an OPT-type transporter located in the plasma membrane, as shown for OPT4 in *A. thaliana* [33]. In fact, the OPT transporter identified in *U. compressa* showed similarity to the OPT transporter detected in the green microalga *Trebouxia* sp. and low similarity to plant OPT. In addition, the increase in transporter activity in response to a sulfide donor may indicate that transporters are activated by persulfidation in *U. compressa*, as has been shown for enzymes glutamine synthase, ascorbate peroxidase, and glyceraldehyde 3-phosphate dehydrogenase in *A. thaliana* treated with H_2_S [34,35,36]. In this sense, OPT-type transporters and others will be characterized in the future in *U. compressa*, along with their potential persulfidation levels.

Regarding the accumulation of Cd in *U. compressa*, the alga cultivated with 10 µM Cd showed electron-dense nanoparticles that do not contain S or N. In this sense, it has been shown that green microalgae such as *C. reinhardtii*, *C. acidophila*, and *Scenedesmus* sp. display electron-dense nanoparticles, probably composed of fatty acids and Cd, where Cd is bound via the carboxyl ends of fatty acids or as CdO, as shown in the green microalga *M. denticulata* [9,10,14]. A transcriptomic analysis showed that *Scenedesmus* sp. redirects carbon flux toward fatty acid and triglyceride synthesis, leading to the inhibition of growth and photosynthesis [11]. The alga treated with Cd and a sulfide donor showed electron-dense nanoparticles without S or N, or with S, which could correspond to CdS nanoparticles, and others containing S and N, probably corresponding to Cd bound to GSH. In this sense, it has been shown that a sulfide donor producing H_2_S increases the synthesis of cysteine, GSH, and PCs by upregulating the expression of enzymes that synthesize these compounds [6]. Thus, it is possible that the sulfide donor exerts the same effect in *U. compressa*, increasing the level of free sulfide and the production of GSH, leading to the binding of Cd to sulfur as well as to GSH. It is important to note that the mechanism of Cd accumulation in the alga differs from that used to accumulate copper, since copper is accumulated exclusively as CuS nanoparticles in the alga treated with copper or with copper and NaHS.

## 4. Materials and Methods

### 4.1. Sampling of U. compressa

Juvenile *U. compressa* were collected in Cachagua (32°34′ S), located in central Chile, from April to November 2024. Algae were placed in a cooler with ice, transported to the laboratory, and cleaned manually. A transcriptome analysis of the alga cultivated with 10 µM copper for 1 d was used to detect transcripts encoding chloroplastic TufA, PsbA, and PsaA corresponding to GeneBank accession numbers PZ492934, PZ492935, and PZ492936, respectively, and these sequences were compared with those of other *U. compressa* present in the NCBI database, corresponding to Gene ID 59422245, 59422242, and 59422235, respectively. The nucleotide sequences of the latter genes showed 98–100% similarity with those of Chilean *U. compressa*, indicating that the alga collected in Chile is indeed *U. compressa*. Algae were sonicated twice for 2 min using an ultrasonic bath (Hilab Innovation Systems, model SK221, Jilin, China) to remove epiphytic bacteria. Algae were maintained in aerated synthetic seawater prepared with 35 g of sea salts in 1 L of distilled water, which contained an undetectable level of Cd (<0.001 µg L^−1^), in a culture chamber at 14 °C with a 12 h light/12 h dark photoperiod. Juvenile algae were cultivated with Cd and with increasing concentrations of NaHS for 0 to 7 d. Algae were cultivated in artificial seawater with 10 µM Cd, and with 50, 100, and 200 µM NaHS. Adult *U. compressa* were collected in Cocholgüe (36°36′ S) in July 2025, cleaned, and maintained in a culture chamber as described above, and used to detect Cd in the alga cultivated with 10 µM Cd and 500 µM hypotaurine for 5 d.

### 4.2. Culture of U. compressa with Cd, NaHS, or Hypotaurine

Algae (10 g of fresh tissue [FT]) were cultivated in 300 mL of artificial seawater (35 g of sea salts in 1 L of distilled water) with 10 µM Cd, and with 10 µM Cd supplemented with 50, 100, or 200 µM NaHS for 1, 3, 5, and 7 d, or with 500 µM hypotaurine for 5 d. After culture, algae were washed twice with 60 mL of 50 mM Tris-HCl–10 mM EDTA to remove Cd from the algal cell wall, allowing only intracellular Cd to be quantified. It is important to mention that this concentration of EDTA did not damage the cell membrane of the marine alga, as observed by confocal microscopy. Algae (9 g FT) were dried in an oven at 60 °C until they reached a constant weight of 700–800 mg of dry tissue [DT], and the levels of intracellular Cd, GSH, and PCs were quantified in algal DT. Algae (1 g FT) were used to quantify transcripts encoding MTs.

### 4.3. Quantification of Intracellular Cd in U. compressa

Algae (500 mg DT) were digested in an Ethos Advanced Microwave Digestion System (Expec Technologies, Hangzhou, Zhejiang, China), and samples were incubated in 9 mL of 65% nitric acid and 1 mL of 30% hydrogen peroxide in Teflon vials at 1800 W for 20 min to reach 210 °C, and then for 15 min at 210 °C. When the temperature reached 30 °C, the vials were placed at 4 °C for 20 min. The vials were then opened, and the final volume was adjusted to 10 mL with distilled water. Intracellular Cd levels were determined by atomic absorption spectroscopy (Analytik Jena, model Nova 350, Jena, Germany; detection limit: 1 µg L^−1^), and the calibration curve was prepared with aqueous solutions containing 0.5, 1, 2, 3, 5, and 7 µg mL^−1^ Cd.

### 4.4. Extraction and Quantification of GSH and PCs in U. compressa

Algae (200 mg DT) were frozen in liquid nitrogen and homogenized in a mortar with a pestle; 1.2 mL of 0.1% trifluoroacetic acid (TFA)–6.3 mM diethylenetriaminepentaacetic acid (DTPA) was added, and homogenization was continued until thawing. The mixture was centrifuged at 14,000 rpm for 20 min, and the supernatant was recovered. The supernatant was filtered through 0.22 µm PVDF filters, and an aliquot of 25 µL was mixed with 45 µL of 200 mM HEPES (pH 8.0) and 6.3 mM DTPA, and 1 µL of 2.5 mM monobromobimane, and incubated at room temperature in the dark for 30 min. The reaction was stopped by adding 30 µL of 1 mM methanesulfonic acid.

GSH and PC levels were determined by high-performance liquid chromatography (HPLC) (Agilent, model 1260 Infinity, Santa Clara, CA, USA), and data were obtained using OpenLab software, version A.01.04.218. An aliquot of 20 µL was analyzed on a reverse-phase C-18 column with 5 µm particles, a 4.6 mm internal diameter, and a 15 cm length (Agilent, Santa Clara, CA, USA) at 24 °C. GSH and PCs were eluted with solvent A (0.1% TFA in aqueous solution) and solvent B (100% acetonitrile) using a linear gradient of 0–20% of solvent B over 10 min, followed by 35–100% of solvent B over 10 min, at a flow rate of 1 mL min^−1^. Pure GSH, PC2, PC3, and PC4 were dissolved in distilled water and used as standards, with retention times of 9, 10, 13, and 16 min, respectively. GSH and PCs were detected using a fluorescence detector with excitation at 380 nm and emission at 470 nm and quantified using calibration curves for each compound ranging from 1 to 50 µM.

### 4.5. Extraction of Total RNA and Quantification of Transcripts Encoding MTs in U. compressa

Algae (100 mg FT) were frozen in liquid nitrogen, pulverized in a mortar with a pestle, and 700 µL of TRK buffer was added. Total RNA was extracted using the E.Z.N.A. Total RNA Kit (Omega Bio-Tek, Norcross, GA, USA) and quantified by absorbance at 260 nm using a microplate reader (Tecan, Zurich, Switzerland). Transcripts encoding MTs and the housekeeping gene β-tubulin were amplified using the PCR primers described in Table 1. cDNA synthesis was performed using 1 µg of total RNA and iScript reverse transcription mix (Bio-Rad, Hercules, CA, USA), completed with DEPC-treated water to a final volume of 20 µL. The solution was incubated for 5 min at 25 °C, 20 min at 46 °C, and 1 min at 95 °C. Amplification of cDNAs encoding MTs and tubulin was performed using 2 µL of cDNA, 5 µL of SsoAdvanced SYBR Green Supermix (Bio-Rad, Hercules, CA, USA), and 1 µL of each PCR primer, in a final volume of 10 µL with DEPC-treated water. The amplification cycles were 40 s at 95 °C, 10 s at 55 °C, and 30 s at 65 °C, using an Aria MX thermocycler (Agilent, Santa Clara, CA, USA).

### 4.6. Analysis of Cd, GSH, and PC Extrusion to the Culture Medium in U. compressa

Algae (10 g FT) were cultivated in 300 mL of artificial seawater containing 10 µM Cd for 5 d, in triplicate. The algae were then transferred to artificial seawater without Cd, and the culture medium was collected after 1, 2, and 3 d of culture. Cd concentration was determined by electrothermal atomic absorption spectroscopy (ET-AAS) [33]. An aliquot of 0.5 mL was filtered through 0.22 µm PVDF membranes, diluted to 5 mL with milliQ water, and analyzed using an ET-AAS instrument (Thermo Scientific, model iCE 300, Whaltman, MA, USA; detection limit: 0.001 µg L^−1^) equipped with a Cd hollow-cathode lamp (Heraeus, Hanau, Germany), with argon as purge gas at a flow rate of 0.2 mL min^−1^. A 50 µg L^−1^ Cd standard solution (Merck, Darmstadt, Germany) was used for calibration, and 10 g L^−1^ magnesium nitrate (Merck, Darmstadt, Germany) was added as a matrix modifier.

Algae (10 g FT) were cultivated in 300 mL of artificial seawater with 10 µM Cd, or 10 µM Cd and 200 µM NaHS, for 5 d in triplicate, then transferred to artificial seawater without Cd, and the culture medium was collected after 1, 2, and 3 d of culture. GSH and PC levels were quantified as described in Section 2.4.

### 4.7. Analysis of Cd Electron-Dense Particles in U. compressa

Algae (2 laminae) were placed in 2 mL of 0.1 M cacodylate buffer (pH 7.2) containing 1% glutaraldehyde and stored at 4 °C. Algae were embedded in epoxide resin, stained with osmium and uranyl acetate, and ultrathin sections of 80 nm were obtained using an ultramicrotome (Leica, Wetzlar, Germany). Sections were placed on copper grids and analyzed by TEM (Thermo Fisher Scientific, model Talos 200 kV, Waltham, MA, USA) coupled to an EDX spectrometer (Quantax Xflash 6T-30, Bruker, Coventry, UK), located at the Center of Biomedical Research, University of Granada, Granada, Spain.

### 4.8. Statistical Analyses

Significant differences were determined by one-way ANOVA followed by Tukey’s multiple comparison test, using the Shapiro–Wilk normality test and GraphPad Prism 8. Differences among mean values were considered significant at *p* < 0.05.

## 5. Conclusions

In conclusion, a sulfide donor increased intracellular Cd levels, and a sulfide acceptor decreased them, suggesting that Cd could be accumulated as CdS in *U. compressa*. GSH levels did not correlate with intracellular Cd levels, and GSH decreased at day 7 compared to day 5, suggesting that GSH may be used to extrude Cd into the culture medium. PC3 and PC4 levels decreased at day 7 compared to days 3 and 5, suggesting that PCs could bind Cd or extrude it into the culture medium; however, their levels were very low compared to those of GSH suggesting that PCs are not involved in Cd extrusion. The increased expression of MT1.1 family members did not correlate with intracellular Cd accumulation, and MT2 was less responsive to Cd than the MT1 family; overall, MT levels did not correlate with intracellular Cd levels. In addition, MT3 did not respond to Cd or to Cd with NaHS. It was shown that Cd was extruded into the culture medium together with GSH, but to a much lesser extent with PCs, and GSH levels were higher than those of Cd (both in µmol L^−1^). Cd was accumulated in the chloroplast, probably bound to fatty acids or as CdO, as observed in microalgae; in response to a sulfide donor, Cd accumulated in the cytosol and chloroplast bound to S, probably as CdS, or bound to S and N, probably complexed with GSH. These findings indicate that the accumulation of copper and cadmium in *U. compressa* occurs through markedly different mechanisms.

## Figures and Tables

**Table 1:** PCR primers for amplification of tubulin and MTs.

Tubulin	5′-TGCAACTTTTGTAGGCAACTC-3′	5′-CAGTGAACTCCATCTCGTCC-3′
MT1.1	5′-CATGGACTGCCGTTGCG-3′	5′-AGCTAGCACTTGCAACCGC-3
MT1.2	5′-CATCATGGATTGCCGCTG-3′	5′-ATCAGCAGCAGCAGCAGC-3′
MT1.3	5′-CATGGACTGCCGTTGCG-3′	5′-AGCTAGTGCGAGTGAGCGC-3
MT1.4	5′-CATGGACTGCCGTTGCG-3′	5′-CATCTAGCGCGAGCGAGC-3′
MT2	5′-ATGGACTGCCGTTGCGAC-3′	5′-TCTTGCAGGCGCAGGTG-3′
MT3	5′-TCTTGTTGTGAAGCCAGTGA-3′	5′-CACAGTTGCATTCTGCGGTT-3′

## Data Availability

Data is available from this online repository: https://doi.org/10.6084/m9.figshare.32554392 (accessed on 14 June 2026).

## Supplementary Materials

The following supporting information can be downloaded at: https://www.mdpi.com/article/10.3390/ijms27125608/s1.
