# Supplementary material for: Cadmium Is Accumulated as Electron-Dense Nanoparticles, Not Bound to Glutathione (GSH), Phytochelatins or Metallothioneins, and Extruded to the Culture Medium with GSH in the Marine Alga Ulva compressa
Source: Int J Mol Sci. 2026 Jun 22;27(12):5608. doi: 10.3390/ijms27125608 (PMC13300706; doi:10.3390/ijms27125608)
Supplement: Supplementary file 1 [file ijms-27-05608-s001.zip › ijms-4328196-supplementary.pdf]

Table S1 - Intracellular accumulation of Cd in response to NaHS and hypotaurine

Mean values of Cd quantified using EAA (µg g-1 DT)					
Days	Control	10 µM Cd	10 µM Cd+ 50 µM NaHS	10 µM Cd+ 100 µM NaHS	10 µM Cd+ 200 µM NaHS
0	0,00	0	0	0	0
1	2,0	0,0	95,5	16,2	61,2
3	0,0	6,7	73,9	50,1	55,3
5	1,9	2,9	65,1	58,7	54,2
7	0,0	22,0	84,9	41,3	70,5

Standard deviation					
Days	Control	10 µM Cd	10 µM Cd+ 50 µM NaHS	10 µM Cd+ 100 µM NaHS	10 µM Cd+ 200 µM NaHS
0	0,00	0	0	0	0
1	0,1	0,0	17,7	1,6	4,7
3	0,0	0,5	4,5	3,1	3,1
5	0,0	0,2	2,5	2,8	4,5
7	0,0	1,4	3,6	0,9	2,1

Mean values of Cd quantified using EAA (µg g-1 DT)		
Treatment	Mean	SD
10 µM Cd	41	13
10 µM Cd + 500 µM Hypotaurine	15	3

Table S2 - Level of GSH in response to Cd and NaHS

Day	GSH without cadmium mg/g DT			Mean	SD
0	3,45	2,65	3,47	3,2	0,47
1	3,25	3,32	3,7	3,4	0,24
3	3,34	3,51	3,23	3,4	0,14
5	3,28	3,01	4,21	3,5	0,63
7	3,29	3,28	3,32	3,3	0,02

Day	GSH 10 µM Cd mg/g DT			Mean	SD
0	3,45	2,65	3,47	3,2	0,47
1	6,59	7,06	1,94	5,2	2,83
3	2,01	1,83	4,48	2,8	1,48
5	4,59	5,73	5,56	5,3	0,62
7	4,84	3,83	3,61	4,1	0,66

Day	GSH 10 µM Cd + 50 µM NaHS mg/g DT			Mean	SD
0	3,45	2,65	3,47	3,2	0,47
1	38,84	38,15	36,49	37,8	1,21
3	28,55	27,9	31,02	29,2	1,65
5	20,17	19,84	20,75	20,3	0,46
7	22,94	22,89	22,86	22,9	0,04

Day	GSH 10 µM Cd + 100 µM NaHS mg/g DT			Mean	SD
0	3,45	2,65	3,47	3,2	0,47
1	1,91	2,2	2,28	2,1	0,19
3	2,3	2,39	3,97	2,9	0,94
5	4,03	3,18	3,28	3,5	0,46
7	2,08	3,2	3,54	2,9	0,76

Day	GSH 10 µM Cd + 200 µM NaHS mg/g DT			Mean	SD
0	3,45	2,65	3,47	3,2	0,47
1	3,81	5,33	3,82	4,3	0,87
3	8,67	8,63	8,52	8,6	0,08
5	14,37	14,14	14,25	14,3	0,12
7	1,33	1,27	1,21	1,3	0,06

Table S3 - Level of PC2 in response to Cd and NaHS

10 µM Cd + 100 µM NaHS					
Day	PC2 µg/g DT			Mean	SD
0	0	0	0	0,0	0,00
1	22,4	28,2	27,8	26,1	3,24
3	32,7	30,5	30,5	31,2	1,27
5	23,4	25,6	5,5	18,2	11,02
7	44,5	24,6	25,9	31,7	11,13

10 µM Cd + 200 µM NaHS					
Day	PC2 µg/g DT			Mean	SD
0	0	0	0	0,0	0,00
1	30,9	46,7	29,2	35,6	9,65
3	32,8	32,9	29	31,6	2,22
5	27,6	21,8	14.6	24,7	4,10
7	0	0	0	0,0	0,00

Tabla S4 - Level of PC3 in response to Cd and NaHS

10 µM Cd + 100 µM NaHS					
Day	PC3 µg/g DT			Mean	SD
0	0	0	0	0,0	0,00
1	98,6	84,4	90,4	91,1	7,13
3	97,3	113,2	147,2	119,2	25,49
5	99,9	103	129,4	110,8	16,21
7	133,9	138,4	299	190,4	94,05

10 µM Cd + 200 µM NaHS					
Day	PC3 µg/g DT			Mean	SD
0	0	0	0	0,0	0,00
1	153,3	184,9	145,7	161,3	20,79
3	323,1	208,9	326,7	286,2	67,00
5	563,9	566,7	565,3	565,3	1,40
7	67,4	87,3	60,4	71,7	13,96

Table S5 - Level of PC4 in response to Cd and NaHS

10 µM Cd + 100 µM NaHS					
Day	PC4 µg/g DT			Mean	SD
0	0	0	0	0,0	0,00
1	305,8	377,5	390	357,8	45,44
3	389,6	386,7	314,4	363,6	42,60
5	392,1	372,9	342,7	369,2	24,90
7	279,1	383,2	402,4	354,9	66,34

10 µM Cd + 200 µM NaHS					
Day	PC4 µg/g DT			Mean	SD
0	0	0	0	0,0	0,00
1	565,8	477,8	578,2	540,6	54,74
3	616,8	629,2	638,9	628,3	11,08
5	418,9	425,7	422,3	422,3	3,40
7	248,1	299,6	297,5	281,7	29,15

Table S6 - Expression of MTs 1.1 family in response to Cd and NaHS

MT 1.1 (10 µM cadmium)					
Time (days)	Relative level			Mean	SD
0	0,7	1,2	1,2	1,0	0,3
1	1,1	0,6	0	0,7	0,4
3	0,2	0,2	0,2	0,2	0,0
5	0,7	0,3	0,4	0,5	0,2
7	0,0	0,0	0,0	0,0	0,0

MT 1.1 (10 µM cadmium + 50 µM NaHS)					
Time (days)	Relative level			Mean	SD
0	0,7	1,2	1,2	1,0	0,3
1	11,9	11,9	12	11,9	0,1
3	24,3	20,0	18,4	19,2	1,1
5	0,5	0,1	0,1	0,2	0,3
7	0,0	0,0	0,0	0,0	0,0

MT 1.1 (10 µM cadmium + 100 µM NaHS)					
Time (days)	Relative level			Mean	SD
0	0,7	1,2	1,2	1,0	0,3
1	1,6	0,7	1	1,1	0,5
3	0,6	1,3	0,8	0,9	0,3
5	0,9	0,4	0,7	0,7	0,3
7	1,1	1,1	0,6	1,0	0,3

MT 1.1 (10 µM cadmium + 200 µM NaHS)					
Time (days)	Relative level			Mean	SD
0	0,7	1,2	1,2	1,0	0,3
1	2,9	3,1	3	2,9	0,2
3	2,1	4,4	3,0	2,5	0,6
5	5,9	9,02	5,2	5,6	0,5
7	1,5	0,9	1,0	1,2	0,3

Table S7 - Expression of MTs 1.2 family in response to Cd and NaHS

MT 1.2 (10 µM cadmium)					
Time (days)	Relative level			Mean	SD
0	0,9	1,2	1,0	1,0	0,2
1	0,4	0,5	0	0,3	0,2
3	0,1	0,1	0,1	0,1	0,0
5	0,8	0,4	0,6	0,6	0,2
7	0,6	0,5	0,6	0,6	0,1

MT 1.2 Cd (10 µM cadmium + 50 µM NaHS)					
Time (days)	Relative level			Mean	SD
0	0,71	1,2	1,2	1,2	0,0
1	22,1	19,9	46,7	21,0	1,5
3	62,8	43,5	43,2	43,4	0,2
5	550,0	546,2	278,9	548,1	2,7
7	601,9	375,7	511,4	556,6	64,0

MT 1.2 Cd (10 µM cadmium + 100 µM NaHS)					
Time (days)	Relative level			Mean	SD
0	0,9	1,2	1,0	1,0	0,2
1	2,1	0,9	1	1,3	0,6
3	0,3	0,5	0,5	0,4	0,2
5	1,0	0,4	0,4	0,6	0,4
7	31,1	27,6	21,8	29,3	2,4

MT 1.2 Cd (10 µM cadmium + 200 µM NaHS)					
Time (days)	Relative level			Mean	SD
0	0,9	1,2	1,0	1,0	0,2
1	0,3	0,4	0	0,4	0,1
3	0,5	0,7	1,2	0,8	0,3
5	13,2	54,8	51,5	53,2	2,3
7	28,0	28,0	21,7	28,0	0,0

Table S8 - Expression of MTs 1.3 family in response to Cd and NaHS

MT 1.3 Cd (10 µM cadmium)					
Time (days)	Relative level			Mean	SD
0	1,1	0,7	1,4	1,1	0,4
1	0,2	0,3	0	0,2	0,1
3	13,2	7,9	11,0	12,1	1,5
5	0,2	0,2	0,9	0,4	0,4
7	1,1	0,5	0,3	0,6	0,4

MT 1.3 Cd (10 µM cadmium + 50 µM NaHS)					
Time (days)	Relative level			Mean	SD
0	0,7	1,2	1,2	1,0	0,3
1	39,4	38,6	35	37,5	2,6
3	87,2	41,3	33,3	37,3	5,6
5	0,8	1,0	0,3	0,7	0,4
7	0,0	0,0	0,0	0,0	0,0

MT 1.3 Cd (10 µM cadmium + 100 µM NaHS)					
Time (days)	Relative level			Mean	SD
0	1,1	0,7	1,4	1,1	0,4
1	3,4	1,4	0	1,7	1,6
3	0,5	9,0	3,9	6,4	3,6
5	0,4	0,4	0,5	0,5	0,1
7	0,9	1,0	1,1	1,0	0,1

MT 1.3 Cd (10 µM cadmium + 200 µM NaHS)					
Time (days)	Relative level			Mean	SD
0	1,1	0,7	1,4	1,1	0,4
1	27,4	17,3	38	27,4	10,1
3	36,8	46,5	41,9	44,2	3,2
5	25,1	27,3	29,4	27,3	2,2
7	2,6	3,6	4,2	3,5	0,8

Table S9 - Expression of MTs 1.4 family in response to Cd and NaHS

MT 1.4 Cd (10 µM cadmium)					
Time (days)	Relative level			Mean	SD
0	0,6	1,5	1,1	1,1	0,4
1	0,8	0,7	0	0,6	0,2
3	6,0	5,1	4,6	4,9	0,3
5	8,7	0,4	1,4	0,9	0,7
7	0,9	7,2	0,8	0,8	0,0

MT 1.4 Cd (10 µM cadmium + 50 µM NaHS)					
Time (days)	Relative level			Mean	SD
0	0,7	1,2	1,2	1,0	0,3
1	3,8	3,5	4	3,7	0,1
3	9,3	6,4	4,4	7,8	2,0
5	0,3	0,2	0,1	0,2	0,1
7	0,0	0,0	0,1	0,1	0,0

MT 1.4 Cd (10 µM cadmium + 100 µM NaHS)					
Time (days)	Relative level			Mean	SD
0	0,6	1,5	1,1	1,1	0,4
1	0,4	0,2	0	0,3	0,1
3	0,6	3,5	2,9	2,0	2,0
5	3,1	2,0	2,2	2,4	0,6
7	2,4	2,3	0,9	1,8	0,8

MT 1.4 Cd (10 µM cadmium + 200 µM NaHS)					
Time (days)	Relative level			Mean	SD
0	0,6	1,5	1,1	1,3	0,3
1	11,4	14,6	12	11,6	0,3
3	22,3	21,4	12,3	21,8	0,6
5	3,2	5,2	3,3	3,3	0,1
7	1,0	1,0	1,3	1,1	0,1

Table S10 - Expression of MTs 2 family in response to Cd and NaHS

MT 2 Cd (10 µM cadmium)					
Time (days)	Relative level			Mean	SD
0	0,5	1,4	1,4	1,1	0,5
1	3,6	2,4	1	3,0	0,8
3	2,8	2,2	1,8	2,5	0,4
5	0,5	0,6	0,5	0,5	0,1
7	32,2	29,7	29,9	30,6	1,4

MT 2 Cd (10 µM cadmium + 50 µM NaHS)					
Time (days)	Relative level			Mean	SD
0	0,1	0,2	0,3	0,2	0,1
1	0,0	0,0	0	0,0	0,0
3	4,1	2,7	1,6	3,4	1,0
5	0,0	0,0	0,0	0,0	0,0
7	0,1	0,0	0,1	0,0	0,0

MT 2 Cd (10 µM cadmium + 100 µM NaHS)					
Time (days)	Relative level			Mean	SD
0	0,5	1,4	1,4	1,1	0,5
1	10,2	7,2	5	7,6	2,5
3	0,9	3,3	2,5	2,2	1,2
5	0,9	1,2	1,3	1,2	0,2
7	1,0	0,8	0,9	0,9	0,1

MT 2 Cd (10 µM cadmium + 200 µM NaHS)					
Time (days)	Relative level			Mean	SD
0	0,5	1,4	1,4	1,1	0,5
1	1,9	2,4	6	2,1	0,4
3	1,3	0,2	0,1	0,5	0,7
5	0,7	1,3	0,7	0,9	0,3
7	0,2	0,2	0,2	0,2	0,0
